# High expression of PDGFA predicts poor prognosis of esophageal squamous cell carcinoma

**DOI:** 10.1097/MD.0000000000025932

**Published:** 2021-05-21

**Authors:** Na Han, Yan-Yan Zhang, Zhong-Mian Zhang, Fang Zhang, Teng-Yuan Zeng, Yi-Bing Zhang, Wen-Chao Zhao

**Affiliations:** a Department of Oncology, The Second Affiliated Hospital of Zhengzhou University; b School of Basic Medical Sciences; c Department of Physiology and Neurobiology, School of Basic Medical Sciences, Zhengzhou University, Zhengzhou, PR China.

**Keywords:** esophageal squamous cell carcinoma, platelet-derived growth factor A, prognosis

## Abstract

Platelet-derived growth factor A (PDGFA), the most known member of PDGF family, plays a crucial role in occurrence and progression of different tumors. However, PDGFA expression and its clinical significance in esophageal squamous cell carcinoma (ESCC) are not clear. The present study aimed to assess the expression and prognostic value of PDGFA in ESCC.

The Gene Expression Omnibus databases (GSE53625, GSE23400, and GSE67269) and fresh clinical samples were employed for detecting PDGFA messenger RNA expression in ESCC. The associations of PDGFA expression with clinicopathological characteristics were evaluated by chi-square test. Kaplan–Meier analysis and Cox proportional hazard regression model were performed to determine the prognostic value of PDGFA in ESCC patients. PDGFA-related signaling pathways were defined by gene set enrichment analysis based on Gene Expression Omnibus databases.

The PDGFA messenger RNA expression was upregulated in ESCC tissues compared with paired adjacent noncancerous tissues (*P* < .05) and was positively correlated with T stage (*P* < .05). Kaplan–Meier survival analysis suggested that ESCC patients with high PDGFA expression were associated with poorer overall survival compared to those with low PDGFA expression (*P* < .05), especially in advanced T stage (*P* < .05). Cox analyses showed that high expression of PDGFA was an independent predictor for poor prognosis in ESCC patients. Gene set enrichment analysis identified 3 signaling pathways (extracellular matrix receptor interaction, focal adhesion, and glycosaminoglycan biosynthesis chondroitin sulfate) that were enriched in PDGFA high expression phenotype (all *P* < .01).

PDGFA may serve as an oncogene in ESCC and represent an independent molecular biomarker for prognosis of ESCC patients.

## 1 Introduction

Esophageal cancer (EC) is one of the most frequent malignancies in the world due to its high incidence. In 2018, 572,034 new EC cases and 508,585 EC-associated deaths were estimated and, as a result, EC ranks 9th in incidence and 6th in mortality among all malignant tumors.^[1]^ Esophageal squamous cell carcinoma (ESCC) and esophageal adenocarcinoma are 2 major histological subtypes of EC and it is well known that ESCC accounts for over 90% of EC cases in East Asian countries and sub-Saharan Africa.^[2,3]^ Most ESCC patients are in late stage at diagnosis due to the lack of early symptoms. Despite improvements in therapy strategy, the prognosis of ESCC patients is still very poor and the 5-year overall survival (OS) after surgery ranges from 26.2% to 49.4%.^[4]^ Therefore, it is necessary to identify new biomarkers and therapeutic targets for ESCC patients.

The family of platelet-derived growth factors (PDGFs) consists of PDGFA, PDGFB, PDGFC, and PDGFD, which are disulfide-bonded to form 4 homodimers and 1 heterodimer.^[5]^ By binding to PDGFα- or β-receptors, PDGFs play a number of critical roles in cell survival, proliferation, migration, and differentiation.^[6]^ Accumulated evidence over the last years demonstrated that upregulation of PDGFA may exert a crucial role in occurrence and development of various tumors. The elevated expression of PDGFA has been reported in various tumor types, such as liver cancer, breast cancer, and oral squamous cell carcinoma.^[7–9]^ Many studies revealed that PDGFA promoted proliferation and invasion of cancer cells^[10–13]^ and served as angiogenic factor in multiple different cancers.^[14–16]^ In addition, numerous clinical studies indicated that high expression of PDGFs was positively associated with clinicopathological parameters including TNM stage, lymph node metastasis, and depth of invasion.^[17–19]^ Although some studies have revealed that PDGFA functioned as an oncogene to promote tumor progression, contradictory findings have been reported regarding the correlation of PDGFA expression with clinical outcomes of patients. PDGFA overexpression is associated with decreased survival in some malignancies, for example, neuroblastomas,^[19]^ osteosarcoma,^[20]^ oral squamous cell carcinoma,^[9]^ and gastric carcinoma,^[18]^ while a few investigations demonstrated the opposite result in patients with nephroblastoma^[21]^ and showed no significant associations between PDGFA and survival in renal clear cell carcinoma.^[22]^ Although PDGFA has been reported to be crucial in different types of cancer, the expression profiles of PDGFA and its prognostic role in ESCC patients remain elusive.

In the present study, the expression of PDGFA at transcriptional level and its associations with clinicopathological parameters in ESCC were investigated, and the prognostic value of PDGFA expression in ESCC patients were analyzed according to the data obtained from Gene Expression Omnibus (GEO). In addition, the biological pathways in which PDGFA may be involved were identified by using gene set enrichment analysis (GSEA).

## 2 Materials and methods

### 2.1 Data collection

Gene expression profiles of GSE53625,^[23]^ GSE23400,^[24]^ and GSE67269^[25]^ were downloaded from the GEO database (GEO, http://www.ncbi.nlm.nih.gov/geo/) in the National Center for Biotechnology Information, which is regarded as a public repository containing gene expression profiles based on microarray. R software was used for GEO data processing. GSE53625, which was based upon the GPL18109 platform (Agilent-038314 CBC Homo sapiens long noncoding RNAs + messenger RNA [mRNA] microarray V2.0), comprised 179 paired ESCC and adjacent normal tissues with follow-up (60–72.6 months) information and clinicopathological parameters, which were shown in Table 1. GSE23400 and GSE67269, which were based on GPL96 platform ([HG-U133A] Affymetrix Human Genome U133A Array), consisted of 53 and 34 matched ESCC and adjacent noncancerous tissues, respectively, and did not contain clinical characteristics and prognostic status. The difference in PDGFA expression between tumoral and nontumoral tissues was analyzed using GSE53625, GSE23400, and GSE67269. In addition, GSE53625 was employed to investigate the clinicopathological significance and prognostic value of PDGFA expression in tumoral tissues.

**Table 1:** Clinical characteristics of patients with ESCC from GSE53625.

Characteristics	Number of sample size (%)
Age (yr)	
≤59	89 (49.7)
>59	90 (50.3)
Gender	
Female	33 (18.4)
Male	146 (81.6)
Tobacco use	
No	65 (36.3)
Yes	114 (63.7)
Alcohol use	
No	73 (40.8)
Yes	106 (59.2)
Tumor grade	
Well	32 (17.9)
Moderately	98 (54.7)
Poorly	49 (27.4)
T stage	
T1 + T2	39 (21.8)
T3 + T4	140 (78.2)
N stage	
N0	83 (46.4)
N1 + N2 + N3	96 (53.6)
Overall survival	
Survival	73 (40.8)
Death	106 (59.2)

### 2.2 Patients and tissue samples

For analysis of PDGFA expression by quantitative real-time polymerase chain reaction (qRT-PCR), paired primary ESCC tissues and adjacent noncancerous tissues were collected from 22 patients who underwent surgical resection from January 2018 to December 2019 at the Second Affiliated Hospital of Zhengzhou University. All the patients were selected based on the following criteria:

(1) patients who were histologically diagnosed as ESCC;
(2) patients who did not receive tumor-related therapies before surgery;
(3) patients who underwent R0 resection.

Patients were excluded according to the following criteria:

(1) patients with other malignancies;
(2) patients with autoimmune diseases.

Resected ESCC tissues and paired normal tissues were preserved in liquid nitrogen immediately. The present study was approved by the Ethical Committee of the Second Affiliated Hospital of Zhengzhou University and was performed after every patient provided written informed consent. The clinical characteristics of ESCC patients are available in Table 2.

**Table 2:** Clinical characteristics for 22 ESCC specimens.

Characteristics	Number of sample size (%)
Age (yr)	
≤56	11 (50.0)
>56	11 (50.0)
Gender	
Female	6 (27.3)
Male	16 (72.7)
Tobacco use	
No	11 (50.0)
Yes	11 (50.0)
Alcohol use	
No	12 (54.5)
Yes	10 (45.5)
Tumor size	
≤5 cm	7 (31.8)
>5 cm	15 (68.2)
Tumor grade	
Well	4 (18.2)
Moderately	8 (36.4)
Poorly	10 (45.4)
T stage	
T1 + T2	4 (18.2)
T3 + T4	18 (81.8)
N stage	
N0	12 (54.5)
N1 + N2 + N3	10 (45.5)

### 2.3 Quantitative real-time polymerase chain reaction

Total RNA was extracted from 22 pairs of ESCC tissues and adjacent noncancerous tissues using Trizol reagent (Takara, Tokyo, Japan). Complementary DNA was synthesized by using PrimeScript^TM^ RT reagent kit (Takara) according to the manufacture's protocol. Oligonucleotide primers for qRT-PCR were as follows: human glyceraldehyde-3-phosphate dehydrogenase (GAPDH)-F: GGAGCGAGATCCCTCCAAAAT, human GAPDH-R: GGCTGTTGTCATACTTCTCATGG; human PDGFA-F: GCAAGACCAGGACGGTCATTT; human PDGFA-R: GGCACTTGACACTGCTCGT. Quantitative RT-PCR was conducted by FastStart Essential DNA Green Master (Roche, Mannheim, Germany). The procedure for qRT-PCR were as follows: 10 minutes at 94°C, followed by 40 cycles of 94°C for 10 seconds, 60°C for 10 seconds and 72°C for 10 seconds. Relative quantification of PDGFA RNA expression was calculated by using 2^−ΔΔCT^ method based on normalization with GAPDH.

### 2.4 Gene set enrichment analysis

GSEA is an analytical method that estimates whether a predefined set of genes exhibits statistically significant difference under 2 biological conditions.^[26]^ GSEA was carried out to explore the signaling pathway associated with PDGFA expression in tumoral tissues of GSE53625, GSE23400, and GSE67269 using GSEA 4.1.0. Tumoral tissues were divided into high expression group and low expression group based on the median value of PDGFA mRNA expression. Annotated gene set of c2.cp.kegg.v6.0.symbols.gmt was used as reference gene set. Thousand permutations for gene sampling were applied for each analysis to ensure the credibility of the results. A gene set with *P* < .05 and false discovery rate <0.25 was considered statistically enriched.

### 2.5 Statistical analysis

Paired Student *t* test was used to examine the difference in PDGFA expression between ESCC tissues and matched adjacent noncancerous tissues. The associations between clinical parameters and PDGFA expression in tumoral tissues were analyzed by chi-square test. ESCC patients in GSE53625 were divided into high-PDGFA (n = 89) and low-PDGFA expression (n = 90) groups according to the median expression value of PDGFA, and Kaplan–Meier analysis and log-rank test were used to compare the OS between the 2 groups. Cox proportional hazard regression model was selected after the proportional hazard assumption was satisfied. Univariate Cox regression analysis was performed to analyze the relationship of OS with clinicopathological parameters and PDGFA expression in ESCC patients. Multivariate Cox regressive analysis was conducted to determine the prognostic value of PDGFA in ESCC patients by including all the parameters with *P* < .15 in univariate Cox regressive analysis. SPSS 21.0 software (SPSS Inc., Chicago, IL) and STATA 15.0 (Stata Corporation, College Station, TX) were used for statistical analysis. The *P*-value less than .05 was considered to be statistically significant.

## 3 Results

### 3.1 PDGFA mRNA levels in ESCC tissues

**Figure 1:**
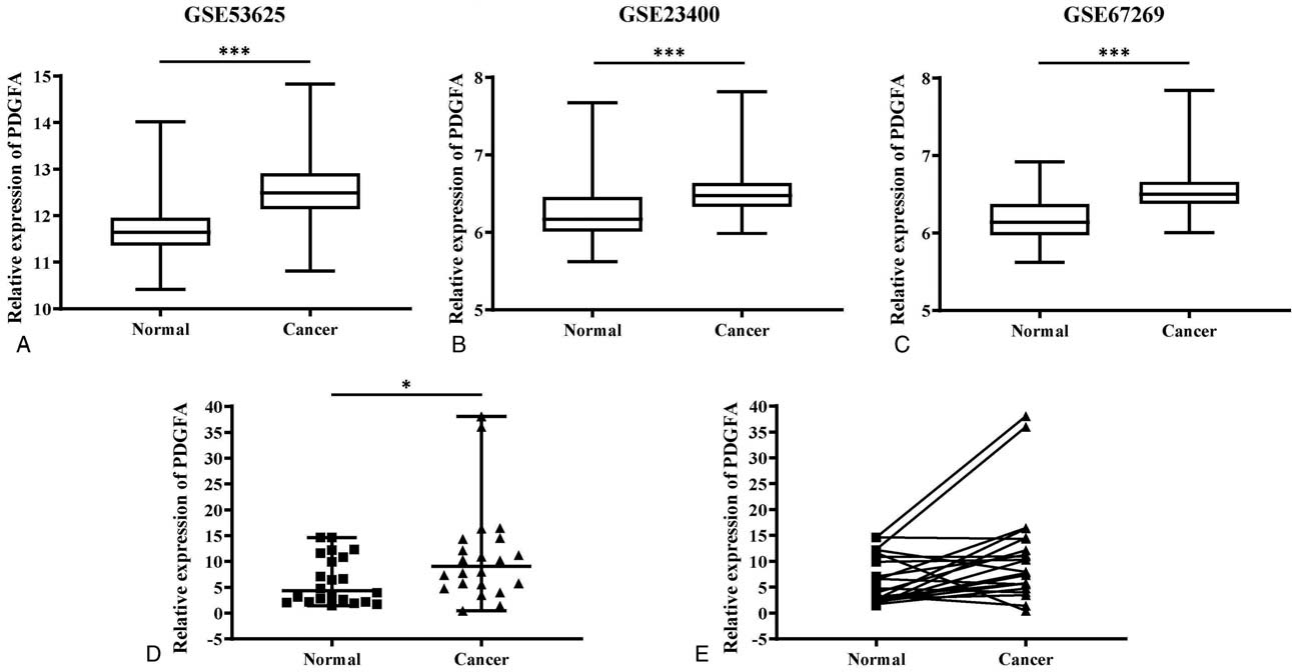
Elevated PDGFA mRNA level in ESCC tissues compared with paired adjacent normal tissues. The expression of PDGFA mRNA in GSE53625 (A) (n = 179), GSE23400 (B) (n = 53), GSE67269 (C) (n = 34), and our study cohort (D and E) (n = 22). (^∗^*P* < .05, ^∗∗∗^*P* < .001). ESCC = esophageal squamous cell carcinoma, mRNA = messenger RNA, PDGFA = platelet-derived growth factor A.

To investigate the role of PDGFA in ESCC, we started with a comparison of PDGFA mRNA levels between ESCC and corresponding normal tissues using the data from GEO database. In datasets of GSE53625, GSE23400, and GSE67269, it was indicated that PDGFA expression was significantly higher in ESCC samples than in adjacent normal tissues (Fig. 1A–C, *P* < .001). To validate the expression of PDGFA in online database, qRT-PCR assay was used to investigate the expression of PDGFA in 22 paired samples from ESCC patients. The results revealed that PDGFA mRNA level was statistically increased in cancer tissues compared to normal tissues (Fig. 1D and E, *P* < .05).

### 3.2 Associations between clinicopathological parameters and PDGFA in ESCC patients in GSE53625

ESCC patients in GSE53625 were divided into high and low groups based on the median expression value of PDGFA in tumoral tissues and the relationship between PDGFA expression and clinicopathological parameters were investigated using chi-square test. As summarized in Table 3, high PDGFA expression was more frequently observed in ESCC patients with more advanced T stage (*P* < .05). However, there were no significant correlations between PDGFA expression and age, gender, tobacco use, alcohol use, tumor location, tumor grade, and N stage (*P* > .05) (Table 3).

**Table 3:** Relationship between PDGFA expression and clinicopathological parameters of ESCC patients in GSE53625.

			PDGFA expression					
Parameters	Groups	N	High	%	Low	%	*χ*^2^	*P*-value
Age (yr)	≤59	89	45	50.0	44	49.4	0.006	.940
	>59	90	45	50.0	45	50.6		
Gender	Female	33	12	13.3	21	23.6	3.134	.077
	Male	146	78	86.7	68	76.4		
Tobacco use	No	65	31	34.4	34	38.2	0.273	.601
	Yes	114	59	65.6	55	61.8		
Alcohol use	No	73	35	38.9	38	42.7	0.269	.604
	Yes	106	55	61.1	51	57.3		
Tumor location	Upper	20	9	10.0	11	12.4	0.517	.772
	Middle	97	51	56.7	46	51.7		
	Lower	62	30	33.3	32	35.9		
Tumor grade	Well	32	13	14.5	19	21.3	2.119	.347
	Moderately	98	49	54.4	49	55.1		
	Poorly	49	28	31.1	21	23.6		
T stage	T1 + T2	39	14	15.6	25	28.1	4.126	.042
	T3 + T4	140	76	84.4	64	71.9		
N stage	N0	83	39	43.3	44	49.4	0.671	.413
	N1 + N2 + N3	96	51	56.7	45	50.6		

### 3.3 PDGFA expression in association with survival of ESCC patients

**Figure 2:**
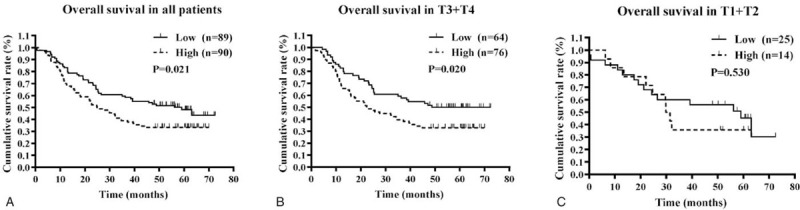
Kaplan–Meier curves of OS of ESCC patients according to PDGFA expression in ESCC tissues. OS analysis for all ESCC patients (A), ESCC patients with high T stage (B), and low T stage (C). ESCC = esophageal squamous cell carcinoma, OS = overall survival, PDGFA = platelet-derived growth factor A.

Through data mining in GSE53625 database, Kaplan–Meier analysis was performed to evaluate the relationship between PDGFA mRNA expression and OS based on the median expression value of PDGFA. The results revealed that ESCC patients with high PDGFA mRNA expression had a poorer OS compared with those with low PDGFA mRNA expression (Fig. 2A, *P* < .05). As shown in Table 3, the differential expression level of PDGFA was statistically associated with T stage, then we further analyzed the association between PDGFA expression level and OS stratified by T stage. Subgroup analysis results suggested that high PDGFA expression was associated with unfavorable OS in patients with advanced T stage (T3 + T4) (Fig. 2B, *P* < .05). However, the association between PDGFA expression and OS was not observed in patients with low T stage (T1 + T2) (Fig. 2C, *P* > .05).

### 3.4 PDGFA expression is an independent prognostic biomarker for ESCC patients

To define whether PDGFA is an independent prognostic factor for patients with ESCC, univariate and multivariate Cox regression analyses for PDGFA expression and clinicopathological parameters were performed using GSE53625 database. The univariate Cox regression analysis revealed that age (hazard ratio [HR], 1.538; 95% confidence interval [CI], 1.047–2.259; *P* = .028), N stage (HR, 2.129; 95% CI, 1.420–3.193; *P* = .000), and PDGFA expression (HR, 1.565; 95% CI, 1.065–2.300; *P* = .023) were prognostic factors affecting the OS of ESCC patients (Table 4). Subsequently, variates with values of *P* < .15 in the univariate Cox models and T stage, which was associated with PDGFA expression, were subjected to multivariate Cox analysis. The results demonstrated that PDGFA expression remained independently associated with OS (HR, 1.536; 95% CI, 1.034–2.282; *P* = .034), as well as N stage (HR, 2.143; 95% CI, 1.400–3.279; *P* = .000) and tumor location (lower vs upper: HR, 0.488; 95% CI, 0.255–0.934; *P* = .030) (Table 5). Taken together, the results indicated that high PDGFA was a poor independent prognostic factor for ESCC patients.

**Table 4:** Univariate Cox regression analyses in patients with ESCC from GSE53625.

Covariate	Hazard ratio (HR)	95% Confidence interval	*P*-value
Age (yr)	1.538	1.047–2.259	.028
>59 vs ≤59			
Gender	0.782	0.489–1.252	.306
Male vs female			
Tobacco use	0.749	0.508–1.104	.144
Yes vs no			
Alcohol use	0.864	0.588–1.269	.457
Yes vs no			
Tumor location			
Upper	1.00 (ref)		
Middle	0.681	0.386–1.203	.186
Lower	0.600	0.326–1.107	.102
Tumor grade			
Well	1.00 (ref)		
Moderately	1.014	0.587–1.749	.961
Poorly	1.652	0.924–2.954	.090
T stage	1.091	0.687–1.732	.713
T3 + T4 vs T1 + T2			
N stage	2.129	1.420–3.193	.000
N1 + N2 + N3 vs N0			
PDGFA expression	1.565	1.065–2.300	.023
High vs low			

**Table 5:** Multivariate Cox regression analyses in patients with ESCC from GSE53625.

Covariate	Hazard ratio (HR)	95% confidence interval	*P*-value
Age (yr)	1.52	1.000–2.256	.050
>59 vs ≤59			
Tobacco use	0.778	0.520–1.165	.223
Yes vs no			
Tumor location			
Upper	1.00 (ref)		
Middle	0.643	0.355–1.165	.145
Lower	0.488	0.255–0.934	.030
Tumor grade			
Well	1.00 (ref)		
Moderately	0.851	0.475–1.525	.587
Poorly	1.234	0.668–2.279	.503
T stage	1.037	0.642–1.677	.881
T3 + T4 vs T1 + T2			
N stage	2.143	1.400–3.279	.000
N1 + N2 + N3 vs N0			
PDGFA expression	1.536	1.034–2.282	.034
High vs low			

### 3.5 Identification of PDGFA-related signaling pathways by GSEA

**Figure 3:**
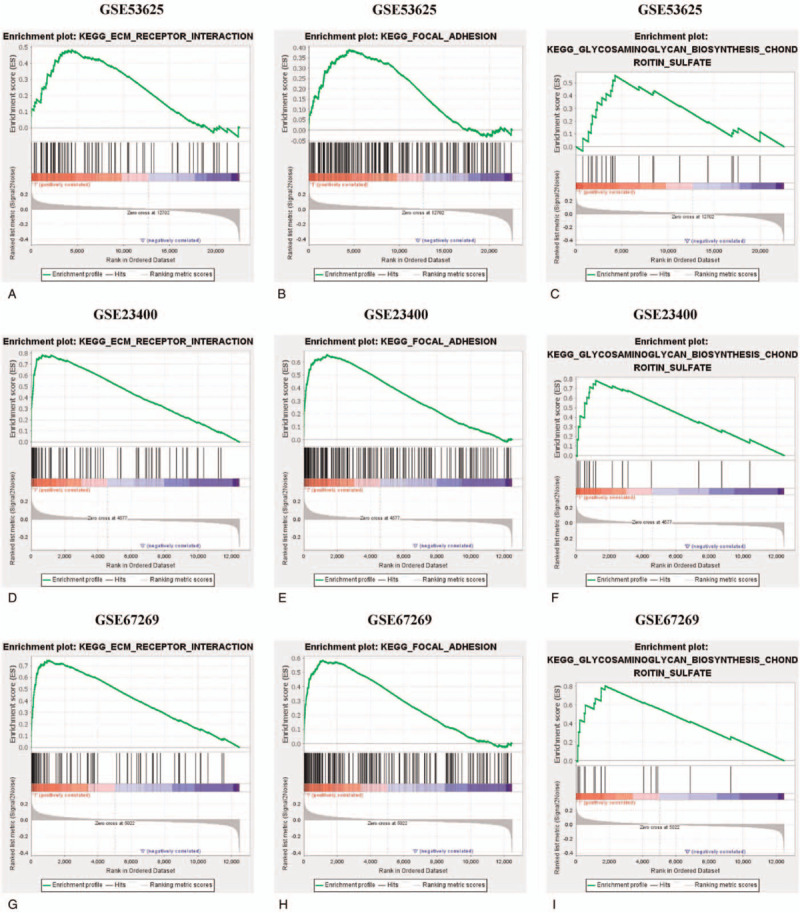
Signaling pathways enriched in high PDGFA phenotype. The GSEA analysis indicated that ECM receptor interaction (A, D, and G), focal adhesion (B, E, and H), and glycosaminoglycan biosynthesis chondroitin sulfate (C, F, and I) were enriched in high PDGFA expression. ECM = extracellular matrix, GSEA = gene set enrichment analysis, PDGFA = platelet-derived growth factor A.

To investigate the signaling pathways associated with PDGFA, GSEA was performed between high and low PDGFA expression datasets based on GSE53625, GSE23400, and GSE67269. The results demonstrated that 3 signaling pathways were significantly enriched in PDGFA high expression phenotype, including extracellular matrix (ECM) receptor interaction, focal adhesion, and glycosaminoglycan biosynthesis chondroitin sulfate, which were shared by GSE53625, GSE23400, and GSE67269 (Fig. 3 and Table 6).

**Table 6:** Signaling pathways enriched in high PDGFA group analyzed by gene set enrichment analysis.

Signaling pathway	ES	NES	NOM *P*-val	FDR *q*-val
GSE53625				
ECM receptor interaction	0.480	2.000	.000	0.006
Focal adhesion	0.390	1.800	.000	0.044
Glycosaminoglycan biosynthesis chondroitin sulfate	0.560	1.690	.008	0.058
GSE23400				
ECM receptor interaction	0.780	2.810	.000	0.000
Focal adhesion	0.660	2.700	.000	0.000
Glycosaminoglycan biosynthesis chondroitin sulfate	0.580	2.050	.000	0.001
GSE67269				
ECM receptor interaction	0.740	2.800	.000	0.000
Focal adhesion	0.580	2.440	.000	0.000
Glycosaminoglycan biosynthesis chondroitin sulfate	0.800	2.160	.000	0.000

## 4 Discussion

As a member of PDGFs family, PDGFA contributes to various of cell processes by activating the corresponding receptors, including cell proliferation, migration, metastasis, and angiogenesis.^[11,27]^ For instance, Cao et al revealed that miR-375 reduced the migration and invasion of oral squamous cell carcinoma cells via targeting PDGFA.^[10]^ Knockdown of forkhead box E1 dramatically promoted proliferation, migration, and invasion of papillary thyroid cancer cells by increasing PDGFA production.^[13]^ Addition of exogenous PDGFA significantly induced cell migration in pancreatic cancer cells.^[11]^ These studies revealed that PDGFA was involved in the progression of human tumors as an oncogene. However, the reports on the association of PDGFA with ESCC are rare. In the present study, a higher PDGFA mRNA expression was observed in ESCC tissues compared with adjacent normal tissues. In addition, it was indicated that the overexpression of PDGFA was significantly associated with advanced T stage and correlated with poor OS. Moreover, 3 signaling pathways related to PDGFA expression were identified.

To our knowledge, elevated expression of PDGFA has been observed in various tumor types, such as head and neck squamous cell carcinoma, renal clear cell carcinoma, liver cancer, and lung cancer.^[28]^ Additionally, Klimczak-Bitner et al found that PDGFA exhibited statistically higher expression level in EC tissues than in the corresponding normal samples.^[29]^ As both ESCC and esophageal adenocarcinoma are main histopathological subtypes of EC, the expression of PDGFA in ESCC is still unknown. Based on the analyses of GEO database, our study showed that the expression of PDGFA in ESCC tissues was significantly higher than that in paired non-cancerous tissues. At the same time, a statistically upregulated expression level of PDGFA was also observed in clinical fresh ESCC tissues compared with matched adjacent normal esophageal tissues by using qRT-PCR. The above results indicated that *PDGFA* gene might function as an oncogene in promoting ESCC tumorigenesis and progression.

Although it has been reported that PDGFA probably contributes to the carcinogenesis and progression of tumors, PDGFA exerts different roles in various types of cancers. The positive correlation between PDGFA expression and N stage was observed in gastric carcinoma, breast cancer, and oral squamous cell carcinoma,^[8,9,18]^ suggesting that PDGFA was a possible accelerator for lymph node metastasis. PDGFA overexpression was associated with higher T stage in papillary thyroid cancer and nephroblastoma,^[13,21]^ implying that PDGFA probably played an important role in the invasion of cancer cells. The possible effects of PDGFA on proliferation of tumor cells was indicated by the positive association of PDGFA expression with tumor size in papillary thyroid cancer.^[13]^ In order to explore the crucial role of PDGFA in ESCC, the clinical value of PDGFA in ESCC was investigated. According to clinicopathological information obtained from GSE53625 dataset, the current study suggested that upregulation of PDGFA was significantly associated with advanced T stage, which was in accordance with the findings in nephroblastoma by Ghanem et al.^[21]^ Obviously, the results about the increased PDGFA in higher T stage implied that PDGFA perhaps possessed the ability to promote the invasion of ESCC cells. This speculation was consistent with several experimental studies on other types of cancers, which demonstrated that PDGFA promoted the invasion of cancer cells in oral squamous cell carcinoma,^[10]^ papillary thyroid cancer,^[13]^ and pancreatic cancer.^[11,30]^ Additionally, Kaplan–Meier analysis showed that high PDGFA expression was associated with unfavorable prognosis ESCC patients, especially in advanced T stage. Subsequent Cox regression analysis indicated that high PDGFA expression was an independent factor to predict unfavorable prognosis, which coincided with the results from a few clinical studies about other kinds of tumors, such as osteosarcoma,^[20]^ nephroblastoma,^[21]^ cholangiocarcinoma,^[17]^ gastric cancer,^[18]^ oral squamous cell carcinoma,^[9]^ and neuroblastoma.^[19]^ Collectively, our investigations suggested that overexpression of PDGFA probably could be used as a prognostic biomarker for ESCC patients. However, Inoue et al found that the expression of PDGFA was not statistically related to the clinical outcomes of ESCC patients,^[31]^ which was inconsistent with our results. This discrepancy might be due to the detection methods of PDGFA and the limited samples in Inoue's study. Therefore, more studies with larger sample size and different detection methods are required for validation of the relationship between PDGFA expression and prognosis in ESCC patients.

To explore the underlying mechanisms responsible for the role of PDGFA, GSEA was performed using GEO datasets. The results of GSEA showed that “ECM receptor interaction,” “focal adhesion,” and “glycosaminoglycan biosynthesis chondroitin sulfate” were significantly enriched in PDGFA high expression phenotype. ECM-receptor interaction, as a micro-environmental pathway for maintenance of cell and tissue structure and function, is upregulated in various types of cancers.^[32–34]^ ECM-receptor interaction plays a crucial role in the cellular processes of cancer cells, such as invasion.^[35,36]^ Focal adhesion pathway exhibits a central role in the interaction between cells. Numerous studies revealed that elevated expression of focal adhesion promoted the invasion of cancer cells.^[37–40]^ As cell surface proteoglycan molecules are involved in cellular recognition and adhesion, glycosaminoglycan biosynthesis chondroitin sulfate pathway is important in modulating cell adhesion, motility, and invasion,^[41]^ which was suggested by the studies about endothelial cells and breast cancer.^[42,43]^ Thus, the GSEA results were in accord with the positive correlation between PDGFA level and T stage, suggesting that the above 3 pathways were possible mechanisms accounting for the role of PDGFA in ESCC. However, the detailed molecular function of PDGFA was not completely defined in the present study and further studies are necessary to elucidate the role of PDGFA in tumorigenesis and progression of ESCC.

Admittedly, there are several limitations in our study. Firstly, the sample size in this research is small. Secondly, some clinical information of ESCC patients were missing, such as disease-free survival and tumor size. Further research with larger sample size and complete clinical information are necessary.

## 5 Conclusion

In conclusion, our study indicated that overexpression of PDGFA was a potential biomarker for poor prognosis of ESCC patients and represented a possible molecular target for the treatment of ESCC. Furthermore, “ECM receptor interaction,” “focal adhesion,” and “glycosaminoglycan biosynthesis chondroitin sulfate” were key pathways associated with PDGFA in ESCC.

## Acknowledgments

The authors sincerely thank the researchers for submitting their microarray original data to GEO database.

## Author contributions

**Conceptualization:** Wen-Chao Zhao.

**Data curation:** Na Han.

**Formal analysis:** Yan-Yan Zhang, Zhong-Mian Zhang.

**Funding acquisition:** Wen-Chao Zhao, Na Han.

**Investigation:** Zhong-Mian Zhang.

**Methodology:** Fang Zhang.

**Project administration:** Wen-Chao Zhao, Na Han.

**Resources:** Teng-Yuan Zeng, Yi-Bing Zhang.

**Software:** Yan-Yan Zhang.

**Supervision:** Wen-Chao Zhao, Na Han.

**Validation:** Wen-Chao Zhao, Na Han.

**Visualization:** Zhong-Mian Zhang, Fang Zhang.

**Writing – original draft:** Na Han.

**Writing – review & editing:** Wen-Chao Zhao.
